# Advances in Janus kinase inhibitors for vitiligo

**DOI:** 10.3389/fimmu.2026.1868416

**Published:** 2026-07-07

**Authors:** Xinyu Wen, Ge Yang, Jing Xue, Lixia Zhang

**Affiliations:** Department of Dermatology, Sichuan Provincial People’s Hospital, School of Medicine, University of Electronic Science and Technology of China, Chengdu, Sichuan, China

**Keywords:** efficacy, Janus kinase inhibitors, pathogenesis, safety profile, vitiligo

## Abstract

Vitiligo is a chronic autoimmune disease that can manifest at any age and affect any anatomical site. Clinically characterized by hypopigmented or depigmented macules and patches on the skin and mucous membranes, and occasionally accompanied by leukotrichia, the condition imposes a profound psychosocial burden and significantly impairs patients’ quality of life. Although conventional therapeutic modalities—including corticosteroids, topical calcineurin inhibitors, phototherapy, and surgical interventions—can achieve varying degrees of disease control, their long-term efficacy is frequently constrained by the chronic, relapsing, and unpredictable nature of the disease.Recently, a deeper understanding of the pathogenesis of vitiligo has highlighted the substantial therapeutic potential of small-molecule targeted agents, particularly Janus kinase (JAK) inhibitors. Emerging evidence from clinical trials and case reports corroborates the efficacy of specific JAK inhibitors in promoting the repigmentation of lesional skin. This review synthesizes current research concerning the mechanisms of action, clinical applications, and safety profiles of representative JAK inhibitors—namely ruxolitinib, upadacitinib, and abrocitinib—in the management of vitiligo, aiming to provide novel insights for future therapeutic strategies.

## 1 Introduction

Vitiligo is a common acquired pigmentary skin disorder, clinically characterized by localized or generalized hypopigmented or depigmented macules and patches on the skin and mucous membranes, occasionally accompanied by leukotrichia. The condition can affect individuals of all ages, with an estimated global prevalence of 0.36% and no sex predilection (1, 2). Vitiligo is frequently associated with an impaired quality of life, exerting a profound impact on emotional well-being, daily activities, and overall psychosocial health (3). Its etiology remains complex and incompletely elucidated, encompassing ongoing controversies; however, prevailing hypotheses implicate a multifactorial interplay of autoimmunity, oxidative stress, genetic susceptibility, and environmental triggers (4). According to the latest global expert consensus issued by the international vitiligo working group in 2023 (5), vitiligo is classified into three principal subtypes: non-segmental, segmental, and undetermined vitiligo.Although conventional clinical modalities, including corticosteroids, topical calcineurin inhibitors, phototherapy, depigmentation therapy, and surgical interventions, are available, their overall efficacy remains limited. Exploring novel therapeutic drug targets through the investigation of pathogenesis is the current mainstream therapeutic strategy for immune-mediated dermatoses. In recent years, with a further understanding of the underlying immune-related mechanisms, small-molecule targeted agents directed at specific targets within the immune-inflammatory pathways of vitiligo, specifically Janus kinase (JAK) inhibitors, have become a novel treatment option, holding promise to provide new possibilities for vitiligo therapy (6). To contextualise this rapidly evolving landscape, we aimed to synthesise current evidence on the pathogenesis of vitiligo and the mechanistic rationale for JAK inhibition. Furthermore, we comprehensively evaluate the latest clinical efficacy, safety profiles, and future applications of three representative JAK inhibitors to provide a robust evidence base for optimising the clinical management of vitiligo.

## 2 Methods

This article is structured as an evidence-based narrative review to provide a comprehensive synthesis of the pathogenesis of vitiligo and the emerging therapeutic landscape of JAK inhibitors targeted therapies. To ensure methodological rigor and transparency, a systematic literature search was conducted across primary electronic databases, including PubMed/MEDLINE, Web of Science, Embase, and the Cochrane Library. Additionally, the ClinicalTrials.gov registry was systematically queried to capture ongoing and completed clinical trials, ensuring the inclusion of the most up-to-date efficacy and safety profiles. The search encompassed literature published from database inception up to May 2026.

The search strategy utilized a combination of Medical Subject Headings (MeSH) and free-text keywords. The core search string was constructed using Boolean operators as follows: (“Vitiligo”[Mesh] OR “vitiligo” OR “non-segmental vitiligo” OR “segmental vitiligo” OR “depigmentation”) AND (“Janus Kinase Inhibitors”[Mesh] OR “Janus Kinase-STAT Signaling Pathway”[Mesh] OR “JAK inhibitors” OR “JAK inhibitor” OR “JAKi” OR “JAK” OR “Janus kinase” OR “JAK-STAT” OR “ruxolitinib” OR “upadacitinib” OR “abrocitinib”) AND (“pathogenesis” OR “mechanism” OR “efficacy” OR “safety” OR “treatment” OR “therapy” OR “adverse events”).

Study selection was rigorously performed based on predefined inclusion and exclusion criteria. Inclusion criteria comprised: (1) peer-reviewed articles published in English; (2) fundamental and translational studies elucidating the molecular pathogenesis of vitiligo; (3) clinical trials (identified by NCT registry numbers), retrospective and prospective cohorts, and high-quality case series evaluating the specific pharmacological focus of ruxolitinib, upadacitinib, or abrocitinib in vitiligo; and (4) comprehensive reviews and meta-analyses providing contextual evidence. Exclusion criteria included: (1) articles published in languages other than English without comprehensive translated abstracts; (2) conference abstracts without full-text access or adequate data; (3) duplicated datasets across multiple publications; and (4) isolated case reports lacking substantial mechanistic or clinical relevance.Any discrepancies between the investigators during the screening process were resolved through consensus or consultation with a senior author.

To maintain the utmost accuracy in data synthesis, all extracted bibliographic data, including PubMed Identifiers (PMIDs) and clinical trial registration numbers, were strictly verified and cross-referenced during the full-text review phase. Ultimately, the selected articles were narratively synthesized to bridge the mechanistic rationale of JAK inhibition with the observed clinical outcomes in vitiligo management.

## 3 Pathogenesis of vitiligo

Vitiligo is an autoimmune disease with a complex etiology, characterized by the targeted destruction of autologous melanocytes under the influence of multifactorial elements, including genetics and the environment. The two principal clinical subtypes, non-segmental vitiligo (NSV) and segmental vitiligo (SV), exhibit fundamentally distinct pathogenic mechanisms. SV is typically characterized by a rapid initial onset, subsequently presenting a unilateral and non-progressive course. Its pathogenesis is closely associated with localized neurogenic mechanisms (such as altered neuropeptide release) and cutaneous somatic mosaicism (post-zygotic mutations rendering specific melanocytes vulnerable), which subsequently trigger a spatially restricted melanocyte-specific cellular immune response (7–9). In contrast, NSV is more common, typically presents with a symmetric distribution, and can affect any anatomical site. Its disease progression is relatively slow and prone to relapse.The development and progression of NSV are primarily co-driven by factors such as oxidative stress, immune responses, genetic susceptibility, and environmental exposure. Investigations into its underlying mechanisms predominantly involve the following five aspects: genetics, oxidative stress, the T helper 1 (Th1) cell pathway, tissue-resident memory T cells, and the WNT signaling pathway (10).

Genetic factors play a significant role in the pathogenesis of vitiligo, and a family history among patients is associated with disease onset. Approximately 15%–20% of patients with vitiligo report having an affected first-degree relative, indicating that genetic factors may contribute to the pathogenesis in certain cases (11).Vitiligo is associated with polygenic inheritance, and genome-wide association studies (GWAS) have identified over 50 vitiligo-associated genes. The genes mapped to these vitiligo-associated loci are involved in antigen presentation, innate immunity, T-cell development and activation, melanocyte homeostasis, melanogenesis, and apoptosis (12, 13).

Oxidative stress is also implicated in the pathogenesis of vitiligo. Various endogenous or exogenous stimuli prompt melanocytes to release reactive oxygen species (ROS) (14). By damaging melanocytes, ROS disrupt melanogenesis and establish a pro-oxidative state within the skin, resulting in localized or generalized cutaneous depigmentation (15). Furthermore, the melanocytes of patients with vitiligo may harbor innate intrinsic structural defects, indeed, culturing melanocytes from the non-lesional skin of these patients is more challenging compared with healthy controls (16).

Patients with vitiligo exhibit a T-cell-mediated autoimmune response, particularly involving a functional imbalance between T helper 1 (Th1) and regulatory T (Treg) cells (17). Driven by this skewed inflammatory milieu, autoreactive CD8+ CTLs are recruited to the skin and serve as the primary effector cells responsible for the direct destruction of melanocytes. Upon recognizing melanocyte-specific antigens, these CD8+ CTLs infiltrate the epidermis, release cytotoxic granules (such as granzyme and perforin), and secrete massive amounts of interferon-γ (IFN-γ) to induce melanocyte apoptosis (18, 19). Within this context, IFN-γ and the JAK/signal transducer and activator of transcription (STAT) signaling pathway play a pivotal role. Beyond the involvement of Th1-associated cytokines and CD8+ CTLs, elevated levels of IL-17 and IL-23 have been detected in the serum and/or lesional skin of patients with vitiligo, suggesting that the Th17 immune axis may also contribute to disease pathogenesis (20). Furthermore, genetic analyses of lesional skin reveal an upregulation of both Th1- and Th2-associated genes, indicating that vitiligo may be driven by diverse immune responses and involve multiple cellular subpopulations (21). Ultimately, these diverse immune responses must transduce signals across the cell membrane via their respective cytokine receptors to exert their intracellular effects, a process that fundamentally relies on the JAK/STAT signaling pathway.

Tissue-resident memory T cells (TRMs) play a crucial role in the recurrence of vitiligo. Following a T-cell-mediated immune response, TRMs, upon stimulation by the key maintenance cytokine IL-15, secrete perforin, IFN-γ, and granzyme B. These mediators exert cytotoxic effects on melanocytes, leading to recurrent depigmentation at the identical lesional sites upon treatment cessation (22–25). Furthermore, studies have demonstrated that in a vitiligo mouse model, treatment with an anti-CD122 monoclonal antibody, which specifically blocks the IL-15 signaling pathway, results in the short-term inhibition of IFN-γproduction by TRMs and the long-term depletion of TRMs from the skin lesions, ultimately facilitating the repigmentation of the depigmented areas (26).

The WNT signaling pathway is essential for the proliferation and differentiation of melanocytes. Oxidative stress suppresses the activation and expression of the WNT pathway in both keratinocytes and melanocytes, leading to impaired melanocyte differentiation and regeneration, which ultimately precludes the repigmentation of lesional skin. In *in vitro* models of vitiligo, treatment with WNT agonists induces the differentiation of follicular and epidermal stem cells into melanoblasts and upregulates the expression of melanocyte markers, indicating that activating this pathway may induce melanocyte regeneration (27).

## 4 Mechanism of action of JAK inhibitors in vitiligo treatment

The JAK-STAT signaling pathway plays a crucial role in the pathogenesis of vitiligo. It serves as a key pathway mediating the signal transduction of various cytokines (including certain interleukins and interferons).Its core components comprise the four members of the JAK family: JAK1, JAK2, JAK3, and tyrosine kinase 2 (TYK2), along with seven STAT proteins (STAT1, 2, 3, 4, 5a, 5b, and 6) (28).

It is important to note that not all cytokines engage the JAK-STAT cascade; rather, this pathway specifically mediates the intracellular signaling of cytokines that bind to type I and type II cytokine receptors. Because these receptors lack intrinsic enzymatic activity, they rely entirely on the associated JAKs to propagate signals (29). Among the complex cytokine network in vitiligo, several key cytokines utilize the JAK-STAT pathway, including IFN-γ, IL-15, IL-23, and IL-6 (30). However, extensive translational and clinical evidence has established that the most critical drivers of disease progression and relapse are IFN-γand IL-15 (26, 31–33). Upon the binding of these specific cytokines to their respective receptors, receptor oligomerization occurs, bringing the receptor-associated JAKs into close proximity to be activated via trans-phosphorylation.These JAKs subsequently phosphorylate specific tyrosine residues on the receptors, creating docking sites for the Src homology 2 (SH2) domains of STAT proteins, leading to their activation. These activated STATs form homo- or hetero-dimers and translocate into the nucleus, where they bind to specific promoter regions—such as gamma-activated sequences (GAS) or interferon-stimulated response elements (ISRE)—participating in the regulation of corresponding gene expression (29, 34, 35).

In terms of immune regulation, two distinct pathways are primarily involved in the complex localized microenvironment. CD8+ CTLs, through the production of IFN-γ, activate JAK1 and JAK2, and induce their autophosphorylation, which in turn activates downstream signal transducers and activators of transcription (specifically STAT1) to regulate intracellular gene expression. This activation process triggers a broader immune crosstalk, specifically, it upregulates major histocompatibility complex (MHC) class I expression on melanocytes to enhance autoantigen presentation, while simultaneously prompting epidermal cells(primarily keratinocytes, acting as potent immune amplifiers) to secrete chemokine (C-X-C motif) ligands (CXCL) 9 and CXCL10. By interacting with their cognate receptor, chemokine (C-X-C motif) receptor (CXCR) 3, CXCL9 and CXCL10 facilitate the recruitment of autoreactive CD8+ T lymphocytes to the epidermis, further exacerbating the inflammatory response and exerting cytotoxic effects (including the release of effector molecules like Granzyme B) on melanocytes (36–38). Concurrently, IFN-γstimulates multiple cell types to produce CXCL10, recruiting lymphocytes to the vicinity of epidermal melanocytes. The CTLs subsequently produce additional IFN-γ, establishing a positive feedback loop that further accelerates melanocyte destruction and leads to cutaneous depigmentation (39). Furthermore, beyond this acute effector phase, the IL-15/JAK1-JAK3/STAT5 axis regulates immune memory. IL-15((trans-presented by its receptor complex) provides critical survival signals((such as upregulating anti-apoptotic proteins like Bcl-2) to CD103+ TRMs in the lesional skin, which are responsible for disease relapse (24–26). Ultimately, this process interconnects inflammatory cytokines and immune cells, resulting in the destruction or dysfunction of melanocytes and driving the formation of depigmented lesions.

**Figure 1:**
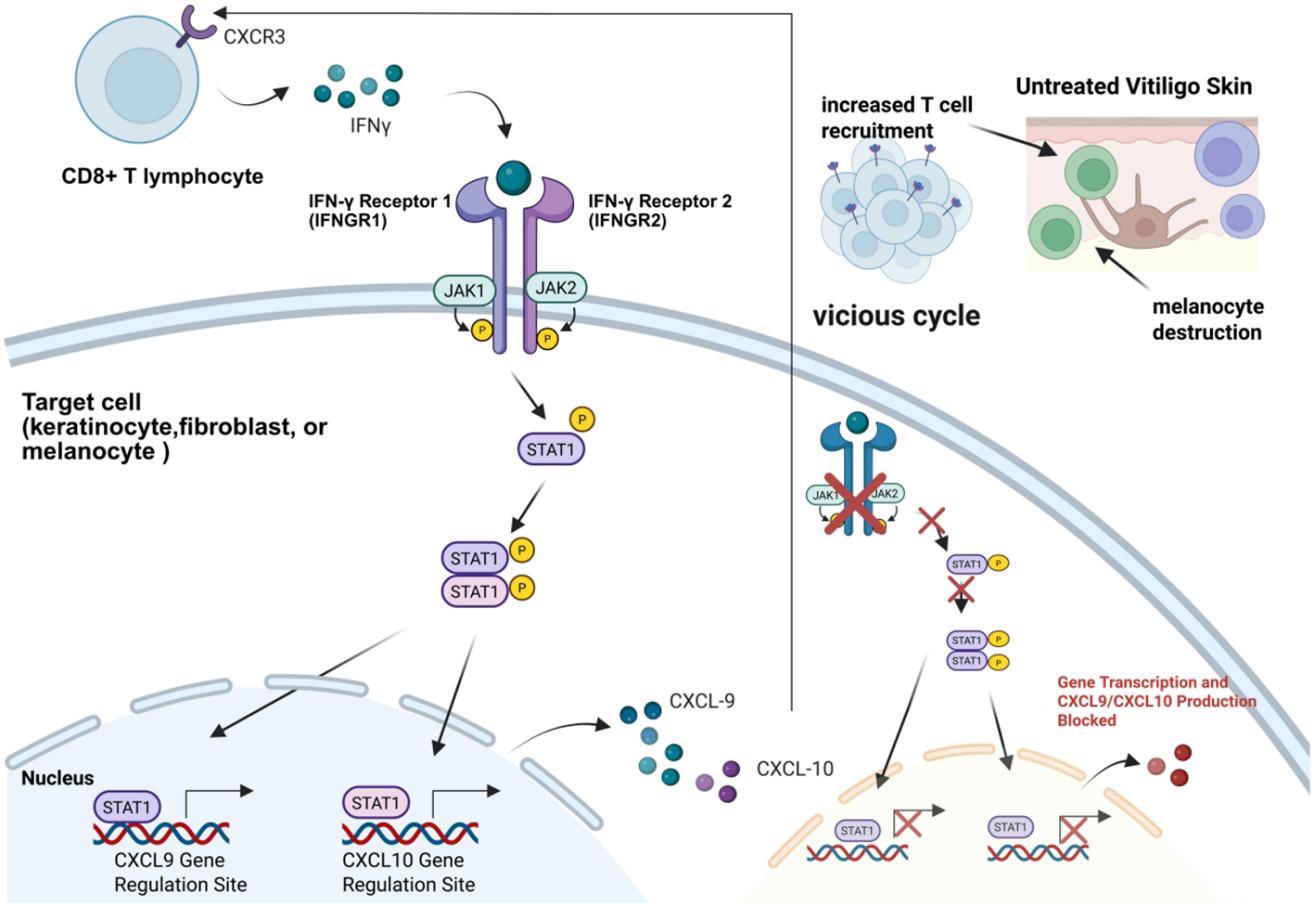
Mechanism of Action of JAK Inhibitors in Vitiligo: Interruption of the IFN-γ-CXCL9/10-CXCR3 axis. Activated CD8^+^T cells (especially skin-resident CXCR3^+^CD8^+^T cells) secrete IFN-γ, which binds to IFNGR1/IFNGR2 on target cells (keratinocytes, fibroblasts, or melanocytes). This activates JAK1/JAK2, leading to STAT1 phosphorylation, dimerization, and nuclear translocation. Nuclear p-STAT1 drives the transcription and secretion of CXCL9 and CXCL10.CXCL9/CXCL10 then recruit more CXCR3^+^CD8^+^T cells to the lesion, amplifying IFN-γ production and forming a vicious positive-feedback cycle. The accumulated CD8^+^T cells destroy melanocytes via granzyme B, Fas-FasL, and IFN-γeffects, resulting in skin depigmentation. JAK inhibitors block JAK1/JAK2, interrupting the cycle and preventing CXCL9/CXCL10 production. Created with BioRender.com.

**Figure 2:**
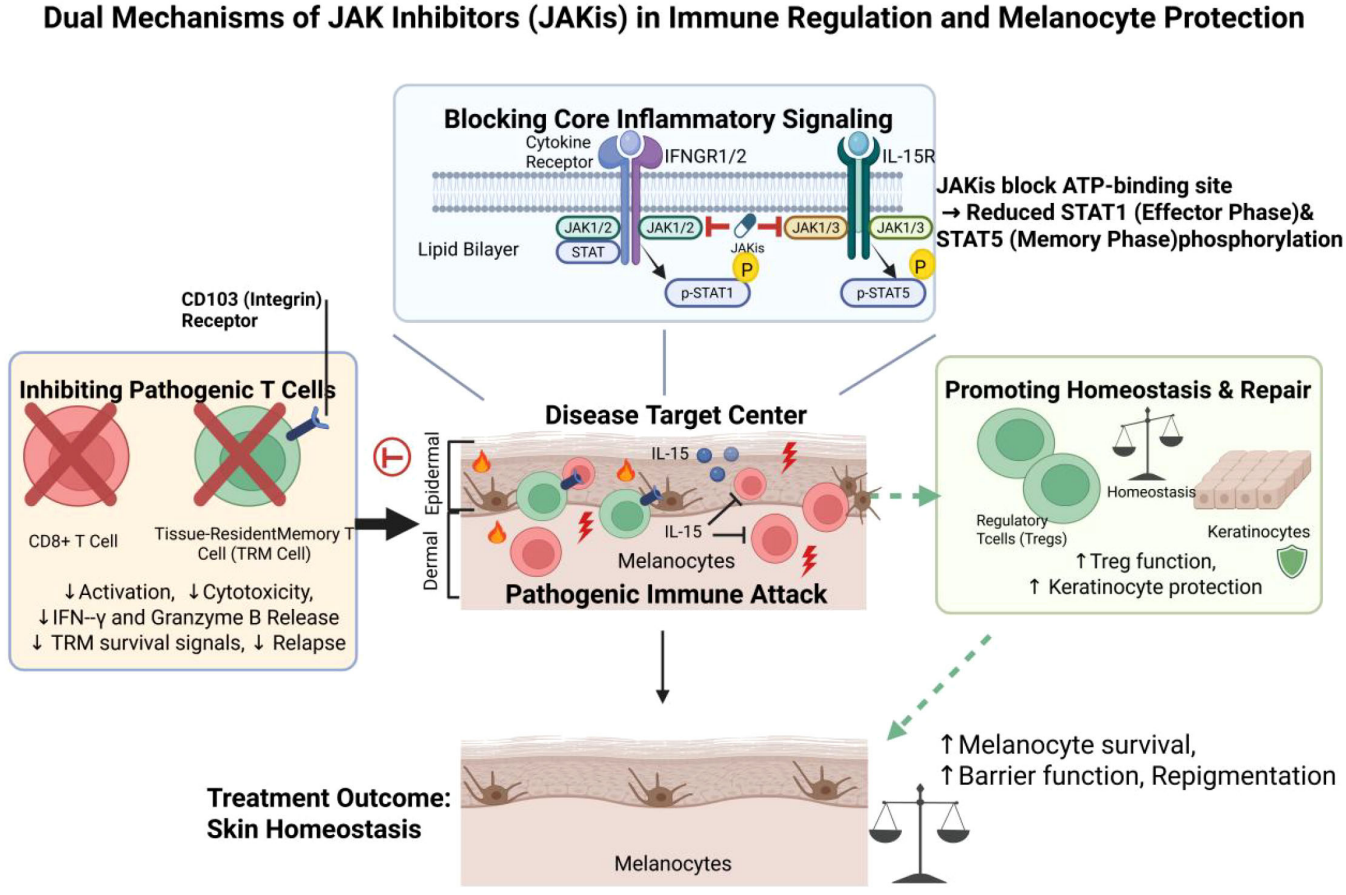
Graphical abstract illustrating the dual mechanisms of JAK inhibitors (JAKis) in modulating both acute and memory phases of immune regulation and melanocyte protection. In the pathogenic microenvironment, autoimmune destruction of melanocytes is driven by acute effector and long-term memory phases. (Top) JAKis competitively block specific Janus kinases, concurrently disrupting two critical signaling axes: the IFN-γ/JAK1-JAK2/STAT1 pathway and the IL-15/JAK1-JAK3/STAT5 pathway. (Left) During the acute effector phase, blockade of the STAT1 axis robustly suppresses the activation and cytotoxicity of pathogenic CD8+ T cells, dampening the release of effector molecules (IFN-γ, Granzyme B). Concurrently, in the memory phase, inhibition of the STAT5 axis deprives CD103+ tissue-resident memory T cells (TRMs) of critical IL-15-dependent survival signals, effectively preventing disease relapse. (Right) Furthermore, JAKis orchestrate microenvironmental repair by enhancing regulatory T cell (Treg) function and conferring protective signals to keratinocytes. (Center & Bottom) Ultimately, this comprehensive dual-phase immune regulation, halting acute melanocyte destruction while preventing long-term recurrence, facilitates sustained melanocyte survival, dermo-epidermal barrier restoration, and stable repigmentation. Created with BioRender.com.

JAK inhibitors are small-molecule targeted agents that suppress the kinase activity of the JAK family by competitively binding to their ATP-binding sites, thereby disrupting the entire downstream signaling pathway and inhibiting the subsequent chemokine cascade (29). Specifically, this blockade prevents the phosphorylation and activation of STAT proteins, consequently precluding their nuclear translocation and the initiation of gene transcription. Within the critical immune signaling pathway of vitiligo, specifically the IFN-γ–CXCL9/CXCL10–CXCR3 axis, IFN-γ produced by CD8+ lymphocytes binds to its specific cell surface receptors, namely IFN-γ receptor 1 and IFN-γ receptor 2. These distinct receptors are constitutively associated with JAK1 and JAK2, respectively (40). By intercepting this signaling axis, JAK inhibitors mitigate the T-cell-mediated destruction of melanocytes and attenuate cytotoxic effects within the cutaneous lesions. Concurrently, by antagonizing the IL-15/JAK1-JAK3 pathway, these agents may disrupt TRM survival, conferring broader immune regulation and potentially erasing localized pathological memory. Furthermore, emerging evidence suggests that JAK inhibition contributes to the restoration of skin homeostasis by promoting the function of regulatory T cells (Tregs) and protecting keratinocyte barrier integrity (32, 41). Together, this multifaceted immune regulation potentially facilitates melanocyte survival and skin repigmentation, thereby representing a highly promising and efficacious targeted therapeutic strategy for vitiligo (26, 38). This signaling pathway and the mechanism of action of JAK inhibitors are illustrated in **Figure 1**. Meanwhile, a comprehensive graphical abstract conceptualizing this dual-phase immune regulation and microenvironmental repair is provided in **Figure 2**.

## 5 Clinical development and application of JAK inhibitors in vitiligo

To date, the development of JAK inhibitors has evolved through three distinct generations. Although first-generation pan-JAK and JAK1/2 inhibitors (such as tofacitinib and ruxolitinib) exhibit remarkable clinical efficacy, their relatively low selectivity and concurrent inhibition of multiple signaling pathways predispose patients to adverse events, including anemia, thrombocytopenia, and an elevated risk of infection. To mitigate these side effects, second-generation highly selective JAK1 inhibitors, represented by upadacitinib and abrocitinib, were developed. By precisely inhibiting pathogenic signaling pathways while preserving the functions of other cytokines, these agents effectively enhance the safety profile of clinical administration. Furthermore, third-generation JAK inhibitors achieve a safer regulatory profile through novel mechanisms, such as allosteric modulation (particularly highly selective TYK2 allosteric inhibitors, represented by deucravacitinib) or covalent binding with dual-target synergy (represented by JAK3 and tyrosine kinase expressed in hepatocellular carcinoma [TEC] family kinase inhibitors, such as ritlecitinib) (42, 43).

This review synthesizes the latest clinical research progress and safety analyses of three representative JAK inhibitors, specifically ruxolitinib (Opzelura^®^, USA), upadacitinib (Rinvoq^®^, USA), and abrocitinib (Cibinqo^®^, USA), in the treatment of vitiligo. The core clinical trial data for these agents are summarized in **Table 1**.

**Table 1:** Trials of emerging JAK inhibitors in vitiligo.

NCT number	Sponsor	Nationality	Phase	Treatment	Drug type	Allocation	Subject	Age	Type	Status	Results	Side effect	Key limitations	References
NCT04896385	Incyte Corporation	The United States, France and Canada	2	Group 1: ruxolitinib cream Group 2: vehicle	JAK1/2 inhibitor (Topical)	Randomized, double-blind, vehicle-controlled	60	≥18	Nonsegmental	Completed	At week 12, a significant decreases in both serum and lesional skin levels of CXCL10.At week 24, a significant increase in melanocyte counts in the basal layer of lesional skin, which was positively correlated with clinical repigmentation (improvement in F-VASI).	Application-site acne and rash/erythema	Small sample size (N = 60); restricted BSA criteria; active lesion bias; lacks long-term clinical durability data.	ClinicalTrials.gov
NCT02809976	Tufts Medical Center	The United States	2	Drug: ruxolitinib 1.5% cream twice daily	JAK1/2 inhibitor (Topical)	N/A	11	≥18	N/A	Completed	At week 20, four patients showed significant repigmentation of the face, and 23% of patients experienced a reduction in their VASI score from baseline.	Only mild side effects	Pilot open-label design; lacks control arm; significant investigator bias; limited generalizability (N = 11).	(50)
NCT03099304	Incyte Corporation	The United States	2	Group 1: ruxolitinib 1.5% cream twice daily Group 2: ruxolitinib 1.5% cream once daily Group 3: ruxolitinib 0.5% cream once daily Group 4: ruxolitinib 0.15% cream once daily Group 5: vehicle	JAK1/2 inhibitor (Topical)	Randomized, double-blind, controlled	157	18 -75	N/A	Completed	At week 24, the 1.5% twice-daily (BID) group had the highest proportion of patients who achieved the primary endpoint (F-VASI50).	Only mild side effects, application-site mild redness, itching or acne	Short-term primary endpoint (24 weeks); dose-ranging design (non-confirmatory); variable anatomical response.	(47, 89)
NCT04052425 (TRuE-V1)	Incyte Corporation	Multicenter	3	Group 1: ruxolitinib 1.5% cream twice daily Group 2: vehicle	JAK1/2 inhibitor (Topical)	Randomized, double-blind, vehicle-controlled	330	≥12	Nonsegmental	Completed	At week 24, 29.8% of patients in the treatment group had achieved F-VASI75 (compared with only 7.4% in the control group). With continued treatment up to week 52, approximately 50% achieved F-VASI75.	Application-site acne, itching, erythema	Highly restrictive inclusion mandates; industry-sponsored endpoint bias; limited evidence for acral/universal disease.	(44, 49, 84, 90)
NCT04057573 (TRuE-V2)	Incyte Corporation	Multicenter	3	Group 1: ruxolitinib 1.5% cream twice daily Group 2: vehicle	JAK1/2 inhibitor (Topical)	Randomized, double-blind, vehicle-controlled	334	≥12	Nonsegmental	Completed	At week 24, 30.9% of patients in the treatment group had achieved F-VASI75 (compared with only 11.4% in the control group). With continued treatment up to week 52, approximately 50% achieved F-VASI75.	Application-site acne, pruritus	Primary focus on facial lesions; insufficient evaluation of real-world comorbidities; lacks long-term safety tracking.	(44, 49, 84, 90)
NCT04530344	Incyte Corporation	Multicenter	3(LTE)	Group 1: ruxolitinib cream	JAK1/2 inhibitor	Open-label	458	≥12	Nonsegmental	Completed	Continued treatment for up to 104 weeks further improves the rate of colour restoration (with some patients achieving F-VASI90)	Application-site mild acne, pruritus	Exploratory mechanism-driven focus; biomarker endpoints outweighing patient-reported quality of life metrics.	(84)
NCT04927975	AbbVie	The United States, France, Japan and Canada	2	Group 1: Upa 22 mg Period 1, then Upa 22 mg Period 2 Group 2: Upa 11 mg Period 1, then Upa 11 mg Period 2 Group 3: Upa 6 mg Period 1, then Upa 6 mg Period 2 Group 4: Placebo Period 1, then Upa 22 mg Period 2 Group 5: Placebo Period 1, then Upa 11 mg Period 2	Selective JAK1 inhibitor (Oral)	Randomised, double-blind, placebo-controlled	185	18 - 65	Non-segmental vitiligo (NSV) and no segmental or localized	Completed	At week 24, a dose-dependent improvement in F-VASI and T-VASI was observed, and 15 mg was identified as the dose offering the optimal balance between efficacy and safety	Upper respiratory tract infections, acne, nasopharyngitis	Narrow population stratification; potential selection bias towards early-stage or specific Fitzpatrick skin types.	(62, 91)
NCT06118411 (Viti-Up)	AbbVie	Multicenter	3(Includes two parallel studies: Viti-Up 1 & 2)	Drug: upadacitinib 15mg oral Drug: placebo Other: NB-UVB (narrow-band ultraviolet B) Phototherapy	Selective JAK1 inhibitor (Oral)	Randomised, double-blind, placebo-controlled	614	≥12	Non-segmented vitiligo	Active (Topline met)	The primary endpoints of T-VASI50 and F-VASI75 were significantly achieved in week 48.	Acne, nasopharyngitis, headache, no serious opportunistic infections (other than herpes zoster).	Single-arm study design; lacks randomization; restricted follow-up period; limited comparative efficacy evidence.	ClinicalTrials.gov

Limitations were assessed based on study design, cohort representativeness, and primary endpoint alignment with clinical practice. “Restrictive BSA criteria” denotes the study exclusion of extensive (>50% BSA) or universal vitiligo. “Active lesion bias” refers to the selective recruitment of rapidly progressing vitiligo to capture accelerated repigmentation signaling, which may limit generalizability to stable disease. “Variable anatomical response” highlights the disparate therapeutic efficacy observed between highly responsive facial lesions and recalcitrant acral segments.For trials without published manuscripts, references are directed to the ClinicalTrials.gov registry. BSA, body surface area; F-VASI, Facial Vitiligo Area Scoring Index; JAK, Janus kinase; LTE, long-term extension; T-VASI, Total Vitiligo Area Scoring Index; Upa, upadacitinib; VASI, Vitiligo Area Scoring Index.

### 5.1 Ruxolitinib

Ruxolitinib is a small-molecule inhibitor that selectively targets JAK1 and JAK2. By blocking the IFN-γsignaling pathway, it significantly downregulates the production of key chemokines, such as CXCL10, by keratinocytes, thereby mitigating cutaneous depigmentation (44). Clinical observations have demonstrated that oral ruxolitinib therapy is accompanied by a rapid decline in serum CXCL10 levels and the repigmentation of lesional skin, directly validating this mechanism of action (45). Furthermore, topical 1.5% ruxolitinib cream achieves elevated drug concentrations within the epidermis and dermis through the highly efficacious local blockade of the JAK pathway, while maintaining exceedingly low plasma concentrations, thereby circumventing the risk of systemic immunosuppression (44). In July 2022, the United States Food and Drug Administration (FDA) approved topical ruxolitinib cream as the sole JAK inhibitor for the treatment of non-segmental vitiligo involving less than 10% of the body surface area in adolescents aged 12 years and older and adults (46).

#### 5.1.1 Established efficacy from high-level evidence

High-certainty evidence derived from these large-scale, double-blind, vehicle-controlled trials establishes topical ruxolitinib as the current standard of care for localized non-segmental vitiligo.A randomized, double-blind, dose-ranging phase II trial (NCT03099304) enrolling 157 adult patients further confirmed that the twice-daily application of the 1.5% cream constitutes the optimal regimen, achieving a ≥50% improvement in the facial Vitiligo Area Scoring Index (F-VASI50) in 58% of patients at week 52 (47). Subgroup analyses revealed that facial repigmentation was the most pronounced and rapid, whereas the therapeutic response in acral regions was relatively modest (47, 48). Two phase 3, randomized, double-blind, vehicle-controlled trials (TRuE-V1 (NCT04052425) and TRuE-V2 (NCT04057573)) demonstrated that at week 24, 29.8% and 30.9% of patients treated with ruxolitinib cream achieved F-VASI75 in TRuE-V1 and TRuE-V2, respectively, compared with only 7.4% and 11.4% in the vehicle groups. With continuous treatment through week 52, approximately 50% of patients achieved F-VASI75, indicating significant repigmentation (44). Additional subgroup analyses demonstrated that the therapeutic response was largely consistent across subgroups stratified by sex, age, and Fitzpatrick skin type, further supporting its broad clinical applicability (49). Notably, while the current phase III trials predominantly enrolled patients aged 12 years and older, phase III studies investigating pediatric patients under 12 years of age are currently ongoing.

#### 5.1.2 Emerging insights from real-world evidence and open-label extensions

Beyond the controlled confines of randomized controlled trials (RCTs), moderate-certainty emerging evidence from open-label extensions and real-world observational cohorts provides valuable insights into the long-term durability and synergistic potential of ruxolitinib.An early open-label study (NCT02809976) demonstrated that the twice-daily application of topical 1.5% ruxolitinib cream significantly improved the F-VASI, with four patients achieving an approximate 76% improvement in the F-VASI and a 23% improvement in the total Vitiligo Area Scoring Index (T-VASI) (50). In a subsequent extension study to 52 weeks, lesions that had previously exhibited no substantial response demonstrated further repigmentation. Notably, the improvement in the T-VASI was more pronounced in the subgroup receiving optional concomitant narrow-band ultraviolet B (NB-UVB) phototherapy, indicating that prolonged application and combination phototherapy may exert a synergistic effect to facilitate the repigmentation of non-facial sites (51). Data from the TRuE-V long-term extension (LTE) study (NCT04530344) indicated that prolonging treatment to 104 weeks conferred further benefits for patients who exhibited limited early responses (52).

Multiple real-world and observational studies have further validated its external validity in clinical practice. Cristallo et al. (53) demonstrated that topical 1.5% ruxolitinib cream induced rapid improvements in both the F-VASI and T-VASI scores among patients with non-segmental vitiligo, achieving an F-VASI75 of 40% at week 12, alongside favorable treatment tolerability and an absence of severe adverse events. In a retrospective analysis of 74 adolescent patients with facial vitiligo, Chen et al. (54) reported a time-dependent improvement in therapeutic efficacy. Specifically, the F-VASI significantly decreased at week 12 and exhibited further improvement at week 24. Adverse events were predominantly mild-to-moderate localized acneiform eruptions, with no severe adverse events or treatment discontinuations reported, thereby confirming the substantial efficacy and favorable safety profile of this agent for adolescent facial vitiligo within a real-world clinical setting.Furthermore, Zhang et al. (55) and Pandya et al. (56) both confirmed that ruxolitinib cream combined with a 308-nm excimer lamp or NB-UVB synergistically facilitates repigmentation, thereby supporting a synergistic repigmentation mechanism driven by the immunomodulatory effects of JAK inhibitors and the UVB-induced stimulation of melanocyte migration.

#### 5.1.3 Hypothesis-generating data from case reports

Finally, low-level evidence from isolated case reports has generated novel hypotheses regarding the extended clinical utility of ruxolitinib.A limited number of case reports have highlighted the potential for rapid onset of action with topical ruxolitinib in pediatric patients, as well as in refractory segmental vitiligo and immune checkpoint inhibitor-induced vitiligo (57–59). Notably, Macit et al. (60) reported a successful repigmentation case utilizing a combination of 5-fluorouracil and topical ruxolitinib. While these clinical outcomes are promising, they also underscore the necessity for vigilance regarding localized adverse events, such as acneiform eruptions or verruca plana.

### 5.2 Upadacitinib

Upadacitinib is a highly selective JAK1 inhibitor, and its oral formulation has been approved by the U.S. Food and Drug Administration (FDA) for the treatment of a spectrum of autoimmune diseases (61). However, its application specifically for vitiligo remains under further investigation, although numerous clinical studies and case reports have demonstrated its therapeutic efficacy. The primary advantage of the oral administration of upadacitinib lies in its ability to cover extensive cutaneous lesions, representing a vital systemic complement to localized therapies.

#### 5.2.1 Advancing high-level evidence (phase ii and phase iii trials)

The clinical efficacy of upadacitinib in non-segmental vitiligo is progressively being established through high-certainty data from large-scale, RCTs. A multicenter, randomized, double-blind, placebo-controlled, dose-ranging phase II trial (NCT04927975) was the first to evaluate its efficacy and safety. The study enrolled 185 patients, who were randomized 2:2:2:1:1 to receive once-daily oral upadacitinib at doses of 6 mg, 11 mg, 22 mg, or placebo during Period 1 (up to 24 weeks). In Period 2 (weeks 24–52), patients in the placebo group were switched to either 11 mg or 22 mg of upadacitinib. Results demonstrated that at week 24, the F-VASI decreased by 21.27% and 19.60% in the 11 mg and 22 mg groups, respectively, while the T-VASI decreased by 10.84% and 14.27%, respectively. Repigmentation across all dose groups continued to improve over time through week 52, with overall favorable tolerability. This trial provided the first evidence that upadacitinib monotherapy can achieve substantial facial and total body repigmentation, particularly for patients with extensive lesions (62). Furthermore, in a subgroup analysis derived from this phase II trial, Ganesan et al. (63) utilized three-dimensional (3D) imaging to demonstrate that the volume of facial pigment recovery in the upadacitinib groups (especially at the 15 mg dose) was significantly superior to that of the placebo group (P < 0.05), providing additional objective support for its therapeutic efficacy.

Two phase III, randomized, placebo-controlled, double-blind studies (the Viti-Up program)(NCT06118411) further evaluated the efficacy and safety of upadacitinib in adults and adolescents (12 years and older) with non-segmental vitiligo. Enrolling a total of 614 patients, the program consisted of Period A (48 weeks), where patients were randomized 2:1 to receive once-daily upadacitinib 15 mg or placebo. Completers subsequently entered Period B, a 112-week open-label extension in which all participants received the 15 mg dose. The 48-week topline results demonstrated that the upadacitinib group met the co-primary endpoints in both studies: T-VASI 50 was achieved by 19.4% in Study 1 (vs 5.9% for placebo) and 21.5% in Study 2 (vs 5.9% for placebo); F-VASI 75 was achieved by 25.2% in Study 1 (vs 5.9% for placebo) and 23.4% in Study 2 (vs 6.9% for placebo). The key secondary endpoint, F-VASI 50, was also significantly superior to placebo. The primary adverse events reported included infections and acne. Long-term open-label extension data (up to approximately 160 weeks) are still being collected and will further clarify the long-term efficacy, durability, and safety of systemic administration.

#### 5.2.2 Emerging real-world evidence in refractory cases

Parallel to ongoing RCTs, emerging moderate-certainty real-world evidence (RWE) from exploratory observational studies highlights its clinical utility, particularly in refractory cases. Zhu et al. (64) retrospectively analyzed five patients with non-segmental vitiligo treated with once-daily oral upadacitinib 15 mg for at least four months. All patients exhibited repigmentation, and peripheral blood analysis revealed a significant decline in CXCL9 levels, a downward trend in the CD4+/CD8+ T-cell ratio, and the downregulation of Th1-like Tregs. Furthermore, a prospective study observed five patients with corticosteroid-resistant vitiligo; after six months of once-daily oral upadacitinib 15 mg, the mean VASI score decreased significantly from 16.77 ± 9.73 at three months to 3.02 ± 17.08 at six months (P = 0.004). Concurrently, serum CXCL10 levels declined from 271.54 to 153.62. Acne occurred in 10% of patients, with no severe adverse events reported. These findings provide prospective real-world evidence for the application of upadacitinib in refractory cases (65).

Further retrospective analyses corroborate these findings. Magdaleno-Tapial et al. (66) retrospectively enrolled 10 patients with non-segmental vitiligo treated with oral upadacitinib (30 mg/day for nine patients and 15 mg/day for one patient). The results showed that 90% of the patients achieved an improvement in their Vitiligo Extent Score (VES), with the mean VES decreasing from 28.6 at baseline to 26 at 24 weeks, demonstrating significant short-term repigmentation without severe adverse events.Furthermore, Su et al. (67) utilized once-daily oral upadacitinib 15 mg to treat 12 patients with recurrent and refractory vitiligo. After at least 16 weeks of continuous therapy, both VASI and Dermatology Life Quality Index (DLQI) scores were significantly improved. Specifically, the mean improvement in pigmentation was 51.4% for the face, 44.6% for the upper limbs and trunk, and 16.8% for the acral regions, indicating a more efficacious therapeutic response in the facial area compared to the acral sites.

Yue et al. (68) reported eight patients with refractory progressive vitiligo treated with oral upadacitinib in combination with NB-UVB, after six months, the mean VASI score decreased from 7.18 to 2.13. Similarly, Yuan et al. (69) retrospectively enrolled 10 adult patients with stable non-segmental vitiligo treated with oral upadacitinib, with some patients receiving concomitant 308-nm excimer laser therapy. The results demonstrated significant clinical repigmentation, which was notably more rapid when combined with phototherapy, suggesting a synergistic effect on repigmentation.Furthermore, Li et al. (70) evaluated the efficacy of oral upadacitinib combined with topical ruxolitinib for progressive non-segmental vitiligo, providing further evidence to support a multimodal combination strategy involving both systemic and topical administration.

#### 5.2.3 Hypothesis-generating data from case reports

Finally, low-level, hypothesis-generating evidence from isolated case reports has documented the successful use of upadacitinib in combination with other therapeutic modalities for the treatment of vitiligo co-occurring with atopic dermatitis (AD) or alopecia areata. This success may be attributed to the overlapping pathogenic mechanisms among these conditions, offering a novel therapeutic option for managing co-existing immune-mediated diseases (71–73). However, as these findings are currently limited to case reports, further large-scale studies are warranted to establish the long-term efficacy and safety of upadacitinib in the treatment of such comorbidities.

### 5.3 Abrocitinib

Abrocitinib, also a highly selective JAK1 inhibitor, has demonstrated its efficacy in AD across multiple phase III clinical trials (74, 75). It is currently approved for the treatment of refractory, moderate-to-severe AD in patients aged 12 years and older who have had an inadequate response to or are ineligible for other systemic therapies, such as corticosteroids or biologics. However, its definitive efficacy and safety profile in the treatment of vitiligo remain under ongoing investigation. Existing literature primarily consists of small-sample prospective observational studies and case reports, with a current lack of support from large-scale phase III RCTs.

#### 5.3.1 Emerging moderate-to-low certainty evidence from observational studies

Currently, moderate-to-low certainty evidence derived from small-sample prospective observational studies provides the initial proof-of-concept for its clinical utility.Xu et al. (76) conducted a prospective observational study involving 11 patients with refractory progressive non-segmental vitiligo. The participants received once-daily oral abrocitinib 100 mg for 16 weeks, followed by a dose reduction to 100 mg every other day (QOD) for an additional 8 weeks in 10 patients, concurrently combined with NB-UVB phototherapy. At week 24, the mean VASI score improved by 22.07%, and 28.6% of patients achieved F-VASI75. Significant declines in CXCL10 and CCL20 levels were also observed. The safety profile was favorable, with adverse events primarily consisting of mild headache and gastrointestinal discomfort, and no severe adverse events were reported. Long-term follow-up at 52 weeks revealed that 72.7% of patients reached T-VASI25 (compared to 54.5% at week 24), indicating well-maintained repigmentation.Notably, patients who showed no response at week 24 might not derive further benefit from prolonged treatment.This study provides the earliest prospective evidence for the real-world application of abrocitinib combined with phototherapy in refractory progressive vitiligo (77).

#### 5.3.2 Hypothesis-generating data from case reports

Furthermore, very low-level, hypothesis-generating evidence from isolated case reports suggests potential off-label applications for complex or recalcitrant cases. Satkunanathan et al. (78) reported a case of a patient with systemic corticosteroid-resistant vitiligo who achieved significant repigmentation after two months of oral abrocitinib 100 mg, suggesting that oral JAK inhibitors may serve as an efficacious option for recalcitrant vitiligo. Additionally, emerging case reports have further highlighted the therapeutic potential of abrocitinib in managing overlapping immune-mediated skin diseases, such as vitiligo co-occurring with AD. Specifically, Shao et al. (79) and Wang et al. (80) documented the successful treatment of refractory AD comorbid with generalized or stable vitiligo. These findings suggest that variations in the JAK inhibition profiles among different inhibitors may influence individual therapeutic responses, warranting future rigorous large-scale validation.

## 6 Safety of JAK inhibitors in vitiligo

Whether administered orally or topically, traditional JAK inhibitors—with the exception of deucravacitinib—are prone to off-target effects due to the high structural homology of their kinase domains. Consequently, these agents carry Black Box Warnings regarding the risks of malignancies, serious infections, major adverse cardiovascular events (MACE), thrombosis, and mortality. Furthermore, warnings exist concerning the potential for cytopenia, gastrointestinal perforation, hepatotoxicity, dyslipidemia, interstitial lung disease, and tuberculosis (35, 81, 82).

Overall, JAK inhibitors demonstrate a generally favorable safety profile in the treatment of vitiligo; however, they present varying risk profiles due to differences in the route of administration and selectivity. First-generation agents are primarily topical formulations with extremely low systemic exposure. Although second-generation oral JAK1 inhibitors offer higher selectivity, close monitoring for JAK-related adverse events, such as infections and acne, remains necessary.

### 6.1 Ruxolitinib

The safety data of topical 1.5% ruxolitinib cream are supported by large-scale phase III clinical trials. Compared with oral Janus kinase inhibitors, topical application results in extremely low plasma concentrations, demonstrating favorable safety and tolerability. One-year post-marketing data by Hu et al. (83) and long-term trials (673 patients) confirmed the absence of major cardiovascular events or mortality (84). The primary adverse events were application-site acne, erythema, pruritus, and hyperpigmentation, most of which were mild to moderate and resolved spontaneously. In very few cases, rare adverse reactions such as myalgia and transient elevations in creatine phosphokinase occurred (85).

### 6.2 Upadacitinib

Phase II and phase III topline results showed that the most frequently reported treatment-emergent adverse events included upper respiratory tract infections, acne, nasopharyngitis, and headache, which were mostly mild. In the Viti-Up 1 study, four cases of serious infections were reported in the upadacitinib 15 mg group, none of which led to the discontinuation of the study drug. Neither of the two Viti-Up studies reported major adverse cardiovascular events, venous thromboembolism, active tuberculosis, or malignancies, and no opportunistic infections other than herpes zoster were reported. The overall safety results were consistent with the known safety profile of upadacitinib, and no new safety signals were identified. A small number of real-world studies reported herpetic gingivostomatitis caused by herpes simplex virus infection and transient, asymptomatic elevations in creatine kinase levels, but no serious adverse events occurred.

Compared with topical JAK inhibitors, oral upadacitinib results in higher systemic exposure, making it more suitable for patients with extensive lesions; however, it requires regular monitoring of laboratory parameters such as complete blood count, blood chemistry, and coagulation profiles.

### 6.3 Abrocitinib

Abrocitinib demonstrates a favorable short-term safety profile in the treatment of vitiligo, with common adverse reactions being primarily mild and transient (such as headache or gastrointestinal reactions), and no serious adverse reactions observed. However, the safety of long-term administration still requires further validation through large-scale randomized controlled trials, particularly regarding the monitoring of infections, thrombosis, and metabolic parameters.

## 7 Discussion

The clinical application of small-molecule JAK inhibitors marks the formal transition of vitiligo treatment from traditional broad immunosuppression to an era of precise targeted regulation of signaling pathways (the IFN-γ/CXCL9-CXCL10 axis). As large-scale phase III trials evaluating highly selective oral JAK inhibitors (e.g., upadacitinib) steadily advance, formal approval is anticipated in the future, providing a more potent systemic therapeutic armamentarium for patients with extensive lesions. Concurrently, the shared pathogenic mechanisms between vitiligo and other autoimmune diseases (such as atopic dermatitis and alopecia areata) may also offer novel insights into the treatment of vitiligo.Current research findings indicate that small-molecule targeted therapies demonstrate significant efficacy in the treatment of vitiligo; however, limitations remain, such as small sample sizes, short follow-up periods, and a lack of control groups, with a general absence of head-to-head comparisons. Moreover, long-term safety data for pediatric and adolescent populations remain scarce, increasing the uncertainty of their use in this demographic. Furthermore, for patients with extensive lesions, how to balance the economic burden of extensive topical application against the systemic risks of long-term oral administration is a practical issue that urgently needs to be addressed.Future research should incorporate larger-sample, multicenter randomized controlled trials, as well as dedicated studies focusing on children and patients with comorbidities, while concurrently conducting long-term real-world follow-up to evaluate the long-term safety of the medications.

Beyond establishing long-term safety profiles, another critical clinical challenge in the chronic management of vitiligo with JAK inhibitors is disease relapse and the durability of repigmentation upon treatment cessation. While JAK inhibitors exhibit remarkable efficacy in suppressing the acute IFN-γ-driven effector phase, clinical observations indicate a substantial rate of depigmentation relapse shortly after discontinuation (47, 86). Mechanistically, this is fundamentally attributed to the persistence of CD103+ TRMs within the lesional skin (38). Although the pharmacological blockade of the IL-15/JAK1/3 axis temporarily deprives TRMs of essential activation and survival signals, it does not induce their apoptosis or physically deplete them from the epidermis. Consequently, upon drug withdrawal, these dormant TRMs rapidly reactivate, driving recurrent depigmentation at the identical anatomical sites (87). This underscores the paradigm that current small-molecule JAK inhibitors function primarily as disease-suppressive rather than curative agents. Therefore, future therapeutic strategies must address this durability bottleneck. Exploring intermittent maintenance dosing regimens to sustain repigmentation, or investigating synergistic combinations with targeted TRM-depleting agents (such as anti-IL-15 or anti-CD122 monoclonal antibodies), represents a crucial frontier for achieving durable, off-drug clinical remission (26).

While overcoming relapse is paramount for sustaining overall disease control, achieving initial repigmentation in specific refractory phenotypes presents an equally pressing hurdle. For instance, compared to sites such as the face and trunk, the efficacy of traditional treatments for vitiligo in acral regions is often suboptimal. Multimodal regimens combined with phototherapy may represent a key direction for optimizing repigmentation in these refractory areas. At the same time, exploring the therapeutic potential of JAK inhibitors in SV remains a critical unmet clinical need. The vast majority of current large-scale trials have exclusively enrolled patients with NSV (44). Due to its localized pathophysiological mechanisms and typical resistance to medical monotherapy, SV is frequently excluded from major studies, leaving high-quality evidence exceedingly scarce (88). Nevertheless, preliminary clinical observations demonstrate that topical JAK inhibitors, particularly when combined with targeted phototherapy (such as the 308-nm excimer laser), can induce significant repigmentation in SV (57, 59). Furthermore, given that surgical epidermal grafting remains a cornerstone for stable SV, investigating JAK inhibitors as an adjunctive therapy to optimize surgical outcomes represents a promising direction for future research (1). Therefore, targeted clinical trials are urgently needed to definitively elucidate their role in this specific subtype. Precise, individualized treatment will undoubtedly bring hope for safer and more highly efficacious repigmentation for vitiligo patients across different subtypes and stages.
